# Processing Information During Regressions: An Application of the Reverse Boundary-Change Paradigm

**DOI:** 10.3389/fpsyg.2018.01630

**Published:** 2018-09-04

**Authors:** Patrick Sturt, Nayoung Kwon

**Affiliations:** ^1^Psychology, School of Philosophy, Psychology and Language Sciences, University of Edinburgh, Edinburgh, United Kingdom; ^2^Department of English Language, Konkuk University, Seoul, South Korea

**Keywords:** eye-movements, reading, regressions, saccades, fixations

## Abstract

Although 10–15% of eye-movements during reading are regressions, we still know little about the information that is processed during regressive episodes. Here, we report an eye-movement study that uses what we call the *reverse boundary change technique* to examine the processing of lexical-semantic information during regressions, and to establish the role of this information during recovery from processing difficulty. In the critical condition of the experiment, an initially implausible sentence (e.g., *There was an old house that John had ridden when he was a boy*) was rendered plausible by changing a context word (*house*) to a lexical neighbor (*horse*) using a gaze-contingent display change, at the point where the reader's gaze crossed an invisible boundary further on in the sentence. Due to the initial implausibility of the sentence, readers often launched regressions from the later part of the sentence. However, despite this initial processing difficulty, reading was facilitated, relative to a condition where the display change did not occur (i.e., the word *house* remained on screen throughout the trial). This result implies that the relevant lexical semantic information was processed during the regression, and was used to aid recovery from the initial processing difficulty.

## 1. Introduction

Although a reader's gaze predominantly moves forwards through the text, approximately 10–15% of eye-movements during reading are regressions (Rayner, 1998). It is well-known that these backward-directed saccades often accompany processing difficulty, but surprisingly little is known about the purpose of regressions, about what type of information is processed during regressive episodes, or about how regressions aid recovery from processing difficulty.

Various proposals have been made about the function of regressions in situations of processing difficulty. In severe cases, the reader may initially fail to integrate the current word into the sentence, and regressions will then allow reprocessing of the previous context, for example, to compute an alternative analysis in the case of a garden path sentence (Frazier and Rayner, 1982). Another possibility is that a new word that poorly fits the context may decrease the reader's confidence about the preceding text, and regressions may then provide a useful means to confirm the relevant properties of the context (Levy et al., 2009; Bicknell and Levy, 2011). Alternatively, as we discuss below, it has been proposed that regressions may serve simply to postpone further input in situations of increased processing load (Blanchard and Iran-Nejad, 1987; Mitchell et al., 2008), in which case, the main purpose of regressions is not to re-read previous input, but to delay moving forward in the text. Of course, it is likely that regressions have multiple functions, possibly including all of the above. For example, the role of regressions in the frequently observed clause wrap-up effect (Rayner et al., 2000; Warren et al., 2009) may well be one of postponing further input, as it is probably related to the need to complete processing of the current clause before moving on to new information in the next clause.

There are two types of empirical questions that may be asked when we examine the function of regressions. One class of questions involves eye-movement control—for example, what is the target of fixations; in which order are words fixated? The second type of question, which is the topic of this paper, is concerned with the information that is extracted during regressions, and how this information is used.

Previous experimental studies on regressions have mainly focused on issues related to eye-movement control, particularly focusing on readers' scanpaths during regressive episodes. The interest in regressive scanpaths is that they can tell us about the strategies that readers use when they encounter processing difficulty, which in turn can act as clues toward the purpose of regressions. For example Mitchell et al. (2008) examined two hypotheses about the strategies that readers use in recovering from the processing difficulty induced by syntactic garden paths. The first hypothesis that Mitchell et al. (2008) examined is the *Selective Reanalysis Hypothesis* (Frazier and Rayner, 1982), according to which the eyes are sent “directly to the ambiguous phrase . . .(i.e., the region containing the information that would permit the parser to locate the source of its error)” (Frazier and Rayner, 1982, p. 188). The second hypothesis examined by Mitchell et al. (2008) is the *Time out Hypothesis*, according to which “the purpose of regressive fixations (and, indeed, re-fixations on the same word) is not to refresh the evidence but merely a delaying tactic used to provide ‘time out’ for as-yet-incomplete parsing operations” (Mitchell et al., 2008, p. 269). Accordingly, Mitchell et al. (2008) reported two eye-tracking experiments that examined regressive eye-movements while readers recovered from syntactic garden paths. In their Experiment 2, they examined garden path sentences like (1a,b).

1.a While those men **hunted the moose** that was sturdy and nimble hurried into the woods and took cover.
1.b One sole hiker spotted that while those men **hunted the moose** hurried into the woods and took cover.

Mitchell et al. (2008) analyzed the distribution of fixations made during regressive episodes following the reader's encounter with the disambiguating verb *hurried*. They observed that this distribution differed in (1.a) and (1.b). Specifically, fixations tended to gravitate toward the words that the authors took to be relevant to recovering from the garden path, namely those words that are highlighted in bold, in both (1.a) and (1.b). This demonstrates that regressive eye-movements are subject to a greater degree of linguistic control than would be predicted by the *Time out hypothesis*. However, as Mitchell et al. (2008) point out, this pattern was found in the context of a more general tendency for the first fixation following a regression to target immediately preceding material, rather than directly landing on linguistically relevant material.

More recent work involving finer-grained analyses of scan paths is also consistent with some degree of linguistic control of regressive eye-movements (von der Marlsburg and Vasishth, 2011, 2013), though this work also shows that there is considerable variability in the strategies that readers employ during regressions.

Although measuring the physical locations of fixations during regressions can tell us a great deal about the strategies that people employ, it is not directly informative about the information that is processed during during regressive episodes, or how that information is used. If regressions are at least partially linguistically controlled, it is expected that fixations during regressive episodes result in the uptake of relevant information, and that this information is directly used in processing.

One way to investigate this issue, which we adopt in the current study, is to make a direct manipulation of the information that is available during regressions. There have been few previous studies that have taken this approach, but we briefly review two of them below. Schotter et al. (2014) directly manipulated the information available during regressions using a *trailing mask* paradigm. In this technique, each word in the sentence is replaced by a mask (i.e., a row of “x”s), when the reader makes a forward saccade out of the word. This means that previous words become unavailable as targets for regressions. Schotter et al. (2014) reported lower comprehension accuracy for conditions where the trailing mask was applied, relative to a natural reading situation. This facilitation was found both for sentences that involved considerable processing difficulty (garden-path sentences), and for unambiguous control sentences. Overall, the results are consistent with the idea that information processed during regressions plays a facilitative role in comprehension.

However, as a similar facilitation effect was found for both garden-path and non-garden-path sentences, the comprehension accuracy results do not tell us specifically about the facilitatory role of regressions in recovery from processing difficulty. Indeed, there are various possible reasons why the trailing mask condition could have reduced comprehension accuracy even in the absence of processing difficulty following a garden path. For example, unlike in natural reading, the trailing mask does not allow the reader to make a regression to a previous word that has been unintentionally skipped. This might have prevented some words from being fully encoded, leading to poorer comprehension accuracy.

Booth and Weger (2013) reported a study that used a contingent-change technique, where a word to the left of fixation was changed during a regressive saccade. An example from their study (Experiment 3) is given in (2):

2 After you clean → paint the table, please remove the waste.

In the procedure used by Booth and Weger (2013), a critical word (e.g., *clean* in 2) changed to another word of the same length (*paint*) when the reader launched a regressive saccade from at least ten characters to the right of that critical word. Follow-up true-or-false questions on each trial probed whether the readers' final interpretation matched the sentence before the display change or after the display change. The authors found that readers' final interpretations matched the post-change version of the sentence, only in cases where the readers re-fixated the critical word during the regressive episode. This indicates that the reader's representation of the word is updated on the basis of fixations made during a regression. While Booth and Weger's (2013) study does suggest that information that is processed during regressions can affect memory of the sentence, as measured by a post-trial comprehension question, it does not directly tell us about how this information affects the reading process itself.

### 1.1. The present study

In the present study, we use a variant of Booth and Weger's (2013) paradigm, which we call the *reverse boundary change technique*, to explore the use of lexical semantic information during regressions, when the reader is recovering from processing difficulty. The study can be seen as a conceptual replication of both Booth and Weger (2013) and Schotter et al. (2014), in the sense that we investigate the degree to which lexical information that is processed during regressions affects the reader's mental representation, and the degree to which such information can be used to facilitate comprehension. At the same time, the study differs from both Booth and Weger (2013) and Schotter et al. (2014), and also extends these studies in several ways. First, we concentrate specifically on the role of regressions in facilitating recovery from processing difficulty—in our case, the processing difficulty was induced using a manipulation of plausibility. The design used by Booth and Weger (2013) did not involve any manipulation of processing difficulty, while the study of Schotter et al. (2014) did not find clear effects on comprehension accuracy as a function of their processing difficulty manipulation. Secondly, while Schotter et al. (2014) and Booth and Weger (2013) rely on evidence from post-trial questions for their main conclusions, we employ on-line measures, directly using the eye-movement record to measure how regressions facilitate recovery from processing difficulty. This allows us to examine not only *whether*, facilitation takes place, but also *when* it takes place, during reading.

A further goal of the experiment reported below is more methodological—we aimed to test the feasibility of the reverse boundary-change technique for investigating the role of regressions in the recovery from processing difficulty. As far as we are aware, ours is the first study that has used the method for this purpose, and we therefore decided to use a relatively simple design in the first instance, in order to establish whether the method is indeed suitable for investigations of this type, and if so, what effects might be expected in the eye-movement record. Thus, it is hoped that this report will be useful for those who are considering using the reverse boundary-change to investigate more detailed and nuanced theoretical questions.

The manipulation that we use in the current study is related to one used by Slattery (2009), who examined the processing difficulty that occurs due to the mis-identification of previously viewed words. Slattery's study examined the mis-identification of words that have high-frequency neighbors (for example, *brunch* has the higher frequency neighbor *branch*), by recording readers' eye-movements while they read sentences like (3a,b):

3a. Due to the freezing rain, the *brunch* was postponed a week.
3b. Due to the freezing rain, the *buffet* was postponed a week.

Note that in (3a.), the initial context of the sentence is compatible both with the critical word *brunch* and its high-frequency neighbor *branch*, while in (3b.), the critical word *buffet* does not have a contextually relevant high frequency neighbor. Slattery (2009) reported more regressions into the critical word for (3a.) than for (3b.), relative to control conditions where the high frequency neighbor was ruled out by the initial context (e.g., *Everyone said the food at the brunch was simply magnificent*). Slattery (2009) interpreted this result as evidence that readers occasionally misidentify *brunch* in (3a.) as its high frequency neighbor *branch*, leading to increased processing difficulty (and thus regressions) when readers reach the final part of the sentence, which is incompatible with the high frequency neighbor [e.g., in (3a.), it is implausible for a *branch* to be postponed].

Our own study was designed to exploit a similar type of processing difficulty, again, in a situation where a potential mis-encoding of a previous word interacts with the plausibility of a sentence. In the context of a potential lexical mis-identification, regressions presumably have a meaningful purpose, namely that of re-checking the (possibly) mis-encoded word, and we therefore expected this to be a suitable test-case of our paradigm.

Specifically, we examined sentences in the following three conditions:

4a. **Plausible:**
  There was an old horse that John had ridden when he was a boy.
  It couldn't run fast any more.
4b. **Change:**
  There was an old house → horse that John had| ridden when he was a boy.
  It couldn't run fast any more.
4c. **Implausible:**
  There was an old house that John had ridden when he was a boy.
  It couldn't run fast any more.

The sentence in (4a-c) is rendered either plausible (4a) or implausible (4c) as a function of which of two lexical neighbors (*horse* or *house*) appears in the context. In the critical *change* condition (4b), the context word *house* is initially presented in the sentence, leading to an implausible interpretation when the reader reaches *ridden*. The context word is then changed to a lexical neighbor (e.g., *horse*) via a display change when the reader crossed an invisible boundary, that appears immediately before the space preceding *ridden* (the boundary marked “|” in 4b). Note that this is similar to the gaze-contingent boundary paradigm (Rayner, 1975; Schotter et al., 2012), except that the display change involves a word to the left of the invisible boundary, instead of a word immediately to the right of the boundary (see also Binder et al., 1999; Apel et al., 2012). We expected that the implausibility of the interpretation in (4b) and (4c) would lead to a high level of regressions launched from the critical word, and from later words in the sentence, relative to the plausible condition (4a). In the *change* condition in (4b), the post-change context word (e.g., *horse*) rendered the sentence plausible. Thus, if readers process and use lexical semantic information during regressions, then we expect processing to be facilitated in the *change* condition (4b), relative to the *implausible* condition (4c).

Within each item, the pre-change (implausible) context word (e.g., *house*) was chosen to have a higher frequency than the post-change (plausible) context word (e.g., *horse*). This was to simulate the situation where readers initially mis-encode a word as a higher frequency neighbor, triggering regressions where the mis-encoded word leads to an implausible interpretation (Slattery, 2009). So, for example, in (4b), at the point where *ridden* is reached, readers should experience processing difficulty, due to the implausible interpretation, leading to an increased probability of regressions. One purpose of these regressions may be to check whether previously encoded words have been mis-perceived (Slattery, 2009). If the reader then checks the context during the regression, a typical situation would be one in which a word in the context is found to be a lower frequency neighbor (*horse*) of the corresponding word encoded in memory (*house*). The *change* condition (4b) was designed to simulate this situation. Note that we do not claim our experiment as a test of this specific explanation of the function of regressions. We merely constructed our stimuli in this way because we believed that this would result in a high proportion of regressions, where these could play a functional role in the reader's recovery from processing difficulty. Large numbers of regressions are needed, in order to allow us to test our hypothesis that lexical semantic information is used in the recovery from processing difficulty, and also to allow us to investigate the feasibility of using the leftward boundary-change paradigm to examine the use of information during regressions.

## 2. Methods

### 2.1. Materials and methods

#### 2.1.1. Participants

Sixty participants from the University of Edinburgh community were paid to participate. Participants were young adults, mostly undergraduate or graduate students. All participants had normal, or corrected-to-normal vision, and were native speakers of English. None of the participants reported having a reading disability. Data for seven additional participants were not included, because of equipment problems resulting in data corruption (*N* = 3), or unresolved calibration difficulties (*N* = 4).

#### 2.1.2. Ethics

The study was approved by the University of Edinburgh's Psychology Research Ethics committee.

#### 2.1.3. Stimuli

Forty-eight stimulus items were constructed, each of which appeared in three conditions, as in (4a,b,c), repeated below (see Appendix in Supplementary Material for a full list of the stimuli):

4a. **Plausible:**
  There was an old horse that John had ridden when he was a boy.
  It couldn't run fast any more.
4b. **Change:**
  There was an old house → horse that John had| ridden when he was a boy.
  It couldn't run fast any more.
4c. **Implausible:**
  There was an old house that John had ridden when he was a boy.
  It couldn't run fast any more.

The items were adapted from those of Slattery (2009). Each experimental item used a pair of context words that differed in the identity of one letter (e.g., *house*/*horse, ankle*/*angle*). Context words were all 4–6 characters long, with a mean of 5 characters. Within each item, the pre-change context word had a higher frequency than the post-change context word [based on the 90 million word written portion of the British National Corpus, the pre-change (implausible) context words had a mean log frequency of 8.96, while the post-change (plausible) context words had a mean log frequency of 6.38].

#### 2.1.4. Norming study

We obtained plausibility ratings for the first sentence of each of the 48 experimental stimuli, in the plausible and implausible conditions. These two conditions were distributed using latin square counterbalancing, and randomly combined with 63 fillers in printed booklets. Twenty-four participants were asked to rate the “naturalness” of each sentence on a scale of 1 (“very unnatural”) to 7 (“very natural”). Fillers included a mix of items that were intuitively plausible and implausible.

Analysis confirmed mean lower ratings for our intended implausible condition (Mean = 2.18, *SE* = 0.32) relative to our intended plausible condition (Mean = 5.88, *SE* = 0.29). A linear mixed effect model using crossed random intercepts and condition slopes for participant and item confirmed that this difference was significant (β = 3.70, *SE* = 0.21, *t* = 17.37).

#### 2.1.5. Procedure

Experimental items were distributed into three lists using a latin-square procedure, and combined with 96 filler items. The stimuli were presented in a pseudo-random order, which was unique for each participant, such that no two experimental items appeared adjacent to each other. One third of all experimental and filler items were followed by a yes-no question, which the participant answered by pressing a left or right button on the response box.

The experiment used an S.R. Research EyeLink 1,000 eye-tracker, with a 21-inch Viewsonic monitor running at a refresh rate of 120 Hz, with a viewing distance of 70cm. Text was presented in black on a white background, using a 14pt Consolas fixed-width font.

The reverse boundary-change procedure was implemented using Eyetrack software developed at UMass^1^. Gaze position was sampled at 1,000 Hz, and the display change was executed when the reader's gaze crossed the invisible boundary. The mean completion time for the display change was 9 ms.^2^

Calibration was done on each participant at the start of the experiment, and a calibration check was carried out after every six trials throughout the experiment, and the participant was re-calibrated as necessary.

### 2.2. Data analysis

Raw fixation data were first screened and corrected for vertical drift. Following screening, any fixation with a duration of less than 80 ms was merged with the previous (or next fixation) if the two fixations were within one character of each other. All remaining fixations less than 80 ms or greater than 1,200 ms were deleted from further analysis.

The stimuli were divided into the following regions for the purpose of analysis:

**Table:** 

**Start of sentence:**	There was an old
**Context word:**	House
**Filler region:**	That John had
**Critical word:**	Ridden
**End of sentence 1:**	When he was a boy.
**Sentence 2:**	It couldn't run fast any more.

The *context word* was analyzed using second-pass reading time, defined as the sum of fixation durations on the word after at least one fixation has been made on the critical word, or later region. We used this measure to test for effects of the word change during regressions—if the word change facilitates recovery from implausibility, then we expect the change condition to show shorter second-pass reading times than the implausible condition. For completeness, we also report first fixation duration on the context word, to give an estimate of initial processing.

The *critical word* was analyzed using three measures (a) first fixation duration (the duration of the first fixation on the word); (b) first-pass regressions (proportion of trials where the first exit from the region was a regression), and (c) go-past time (the sum of fixations from the first fixation on the word until a subsequent region of text was fixated). Here, first-fixation duration and first-pass regressions are intended to measure the initial plausibility effect, before the reader has been able to re-fixate the context word—we expect both the change and implausible conditions to show an equal processing cost relative to the plausible condition in these measures. Note that there is a considerable literature documenting early plausibility effects in reading, with several studies showing increased fixation times (e.g., Rayner et al., 2004; Warren and McConnell, 2007; Matsuki et al., 2011), and other studies also showing increased rates of regression (e.g., Pickering and Traxler, 1998), at the point where a sentence becomes implausible, relative to a plausible control. The effect can be measured as early as the first fixation on the target word (Warren and McConnell, 2007; Matsuki et al., 2011).

In contrast to first-fixation and proportion of regressions, go-past time includes fixations made during regressive episodes to the left of the word, and so could potentially indicate an effect of the changed word; for example, if the changed word facilitates recovery from implausibility, then we expect shorter go-past durations in the change condition relative to the implausible condition. Go-past time on the critical word is the earliest measure that could indicate a change facilitation effect. Such an effect on this word would indicate that reading had been affected by the changed context word following a regression out of the critical word, but before any subsequent regions had been fixated. This would therefore be evidence that the word-change information has a relatively immediate facilitatory effect on processing.

The final two regions (*End of sentence 1; Sentence 2*) were analyzed using *go-past* and *first-pass regressions*. These measures both give information about the degree of continuing difficulty as readers progress through the text, and could both be affected by the word change in the *change* condition, and could thus provide evidence for a relatively delayed change-facilitation effect.

Before conducting the analyses, we excluded trials according to three criteria, given below:

1. We excluded trials from all further analysis where the reader fixated the critical word or subsequent regions without having previously fixated the context word (619 trials, 21% of the total). This was done to ensure that readers would fixate the word both before and after the display change, in cases where they regressed back to the context word after crossing the boundary in the change condition. This criterion was applied equally to all three experimental conditions.
2. We excluded trials from all further analysis in the change condition if the following two conditions applied to any given trial: (a) the display change erroneously occurred before the critical word was fixated^3^, and (b) the context word was fixated after the display change, but before the critical word was fixated (9 trials, < 1% of the total). These trials are not informative about the role of regressions in processing difficulty, because readers fixate the changed context word before receiving the relevant plausibility information from the critical word.
3. After criteria (1) and (2) above had been applied, if a measure returned no relevant fixations for a given region, the relevant data point was treated as missing data, rather than contributing a zero value to the analysis. For example, for first fixation, go-past, and regressions out, the data point was excluded if the region was skipped, while for second-pass reading time, the data point was excluded if it received no second pass fixation. This final criterion applied at the individual region level only (for example, if region N was skipped, this did not affect the analysis of region N+1, with the exception of trials affected by skipping of the context word in relation to criterion 1 above)^4^.

All duration-based measures were log-transformed prior to analysis. Eye-movement measures were then analyzed using Linear Mixed Effect (LME) regression (using a logit link function in the case of first-pass regressions out). The LME model used the implausible condition as the reference level, and included the contrast of this reference condition with each of the two other conditions. The contrast of Implausible vs. Plausible, which we will call the *plausibility effect*, is effectively a manipulation check, to verify that effects of plausibility are detectable using our design. The contrast of Implausible vs. Change, which we will call the *facilitation effect* measures the facilitation afforded by the changed word, as compared with a baseline that does not incorporate the change. The LME models included random intercepts for participant and item, as well as random slopes for subjects and items based on the condition factor. To facilitate convergence, the random effect structure did not include correlation parameters. Convergence was achieved in all cases except for the model for first-pass regressions out of the “end of sentence 1” region, where the item-based random slope parameter was removed. Coefficients were considered to be significantly different from zero if the absolute *t*-value exceeded 2.

### 2.3. Results

**Figure 1:**
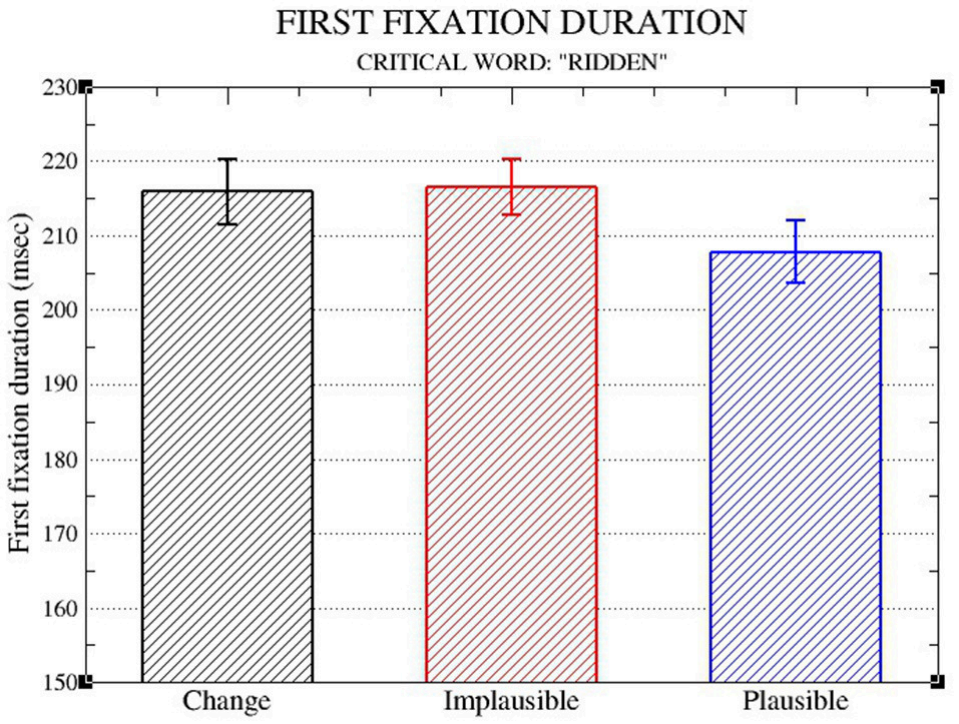
Means and standard errors (aggregated by participant) for First fixation durations on the critical word.

Figure 1 shows the means for First-fixation duration on the critical word, and Table 1 shows the LME results.

**Table 1:** LME results for first fixation duration.

**First fixation duration:**			
**Critical word** ****(ridden)****			
	**Estimate**	***SE***	*t***-value**
(Intercept)	5.332049	0.019862	268.45^*^
Implausible vs. change	−0.002472	0.015928	−0.16
Implausible vs. plausible	−0.042607	0.015788	−2.70^*^

* *p < 0.05*.

The analysis of First Fixation duration confirms an early plausibility effect on the critical word, with longer durations in the implausible condition than the plausible condition, with no significant facilitation in the change condition (Implausible: 216 ms; Plausible: 208 ms; Change: 216 ms). This result is expected, given that the first-fixation duration on the critical word measures what happens after the reader has encountered the plausibility information, but before any regressions to the context word could have taken place.

**Figure 2:**
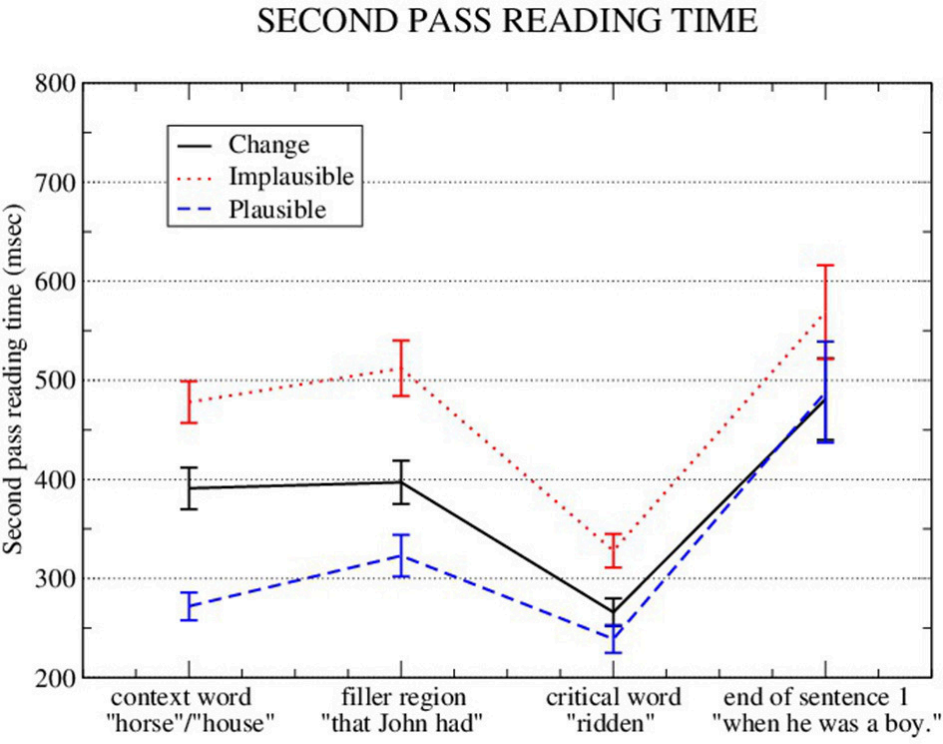
Means and standard errors (aggregated by participant) for Second Pass reading times on the context word, plus the two next regions.

Figure 2 shows the means for Second Pass reading time, and Table 2 shows the LME results for the context word *house/horse*^5^.

**Table 2:** LME results for second pass reading time.

**Second pass reading time**			
**Context region** ****(horse)****			
	**Estimate**	***SE***	*t***-value**
(Intercept)	5.91505	0.04253	139.09^*^
Implausible vs. change	−0.16079	0.03829	−4.20^*^
Implausible vs. plausible	−0.55669	0.05185	−10.74^*^

* *p < 0.05*.

Second-pass reading times at the context word showed both a plausibility effect, and a change facilitation effect (Implausible: 452 ms; Plausible: 265 ms; Change: 369 ms). For completeness, First-fixation durations for the context word were also analyzed, and the means were: Implausible: 204 ms; Plausible: 208 ms; Change: 200 ms; Implausible vs. Change: β = 0.021, *t* = −0.53; Implausible vs. Plausible: β = 0.021, *t* = 1.32.

**Figure 3:**
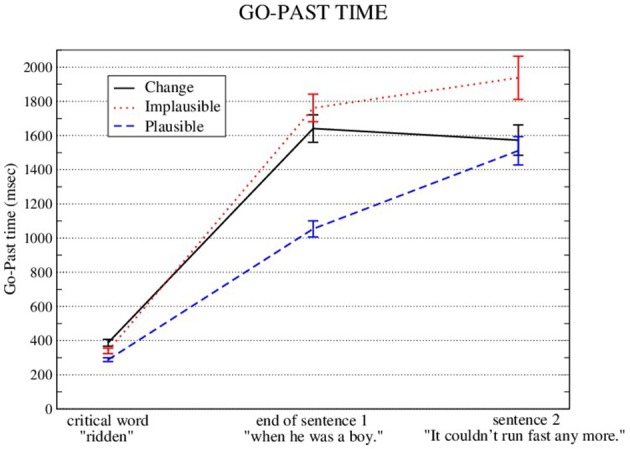
Means and standard errors (aggregated by participant) for Go-Past times on the critical word, plus the next two regions.

**Figure 4:**
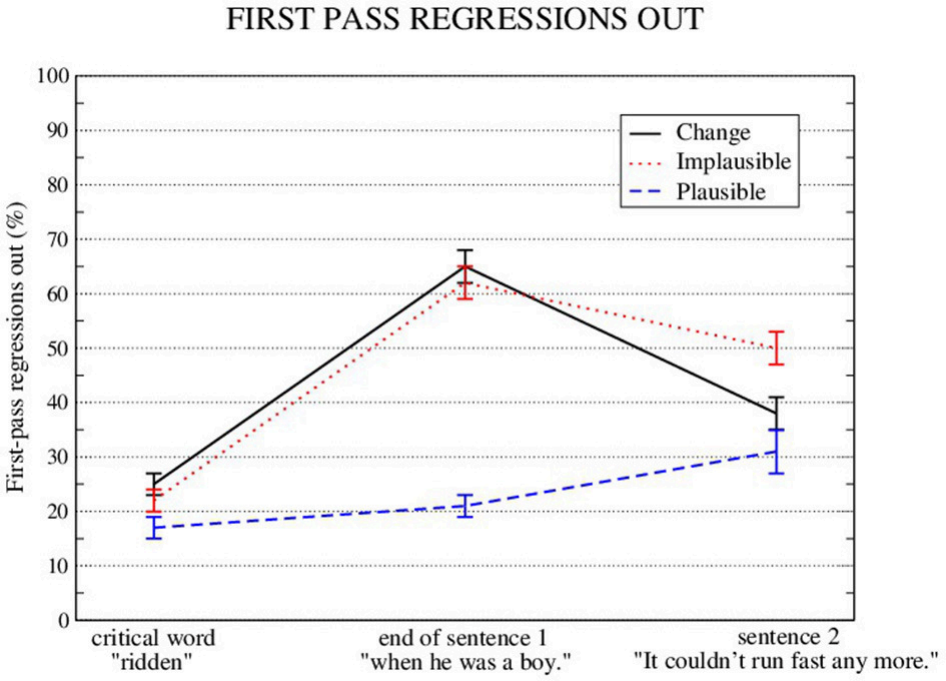
Means and standard errors (aggregated by participant) for First-pass regressions out of the critical word, plus the next two regions.

Go-past times and first-pass regressions out were analyzed for three regions, namely the *critical word* region, the *end of sentence 1* region, and the *sentence 2* region. These measures showed a significant plausibility effect in all three regions (see Figures 3, 4 and Tables 3, 4). They also show a significant change-facilitation effect in the *sentence 2* region (Go-Past: Implausible: 1938 ms; Plausible: 1511 ms; Change: 1573 ms; First-pass regressions: Implausible: 50%; Plausible: 31% ms; Change: 38%).

**Table 3:** LME results for go-past time.

**Go-past time:**			
**Critical word** ****(ridden)****			
	**Estimate**	***SE***	*t***-value**
(Intercept)	5.63246	0.03828	147.12^*^
Implausible vs. change	0.05534	0.03584	1.54
Implausible vs. plausible	−0.12856	0.03006	−4.28^*^
**End of sentence 1** ****(when he was a boy)****			
	**Estimate**	***SE***	*t***-value**
(Intercept)	7.26264	0.05295	137.15^*^
Implausible vs. change	−0.03893	0.02829	−1.38
Implausible vs. plausible	−0.46506	0.03502	−13.28^*^
**Sentence 2** ****(It couldn't run fast anymore)****			
	**Estimate**	***SE***	*t***-value**
(Intercept)	7.34466	0.05348	137.34^*^
Implausible vs. change	−0.16761	0.02422	−6.92^*^
Implausible vs. plausible	−0.20301	0.03099	−6.55^*^

* *p < 0.05*.

**Table 4:** LME results for first-pass regressions out.

**First-pass regressions out:**			
**Critical word** ****(ridden)****			
	**Estimate**	***SE***	*Z***-value**
(Intercept)	−1.4542	0.1604	−9.066^*^
Implausible vs. change	0.1826	0.1430	1.277
Implausible vs. plausible	−0.3441	0.1507	−2.284^*^
**End of sentence 1** ****(when he was a boy)****			
	**Estimate**	***SE***	*Z***-value**
(Intercept)	0.5894	0.1625	3.627^*^
Implausible vs. change	0.1220	0.1205	1.012
Implausible vs. plausible	−2.1901	0.1576	−13.893^*^
**Sentence 2** ****(It couldn't run fast anymore)****			
	**Estimate**	***SE***	*Z***-value**
(Intercept)	0.05586	0.18274	0.306
Implausible vs. change	−0.66241	0.14143	−4.684^*^
Implausible vs. plausible	−1.09409	0.13025	−8.400^*^

* *p < 0.05*.

#### 2.3.1. Debriefing and awareness of the word change

During follow-up debriefings at the end of the experiment, participants were asked whether they noticed anything strange in the experiment, and all participants, when prompted, confirmed that they noticed that a word sometimes had changed to a different one the second time they looked at it. In many cases, participants reported the impression that they had initially misread the word. No participants reported being aware of the actual process of the word changing on the screen. Participants were also asked to estimate the proportion of trials on which they believed that a word change had occurred. Estimates ranged from 2 to 60%, with a mean of 22%^6^. Given that each participant was exposed to 16 change items out of a total of 144 stimuli (including fillers), the maximum proportion of trials on which a participant could potentially be aware of a word change is 11%. Thus, participants tended to over-estimate the proportion of word changes. Participants' estimates were not significantly correlated with the proportion of trials in which each participant actually re-fixated a changed word after crossing the boundary (Spearman's ρ = −0.06). Overall, the participants' estimates appear to be rather inaccurate, and probably do not provide much useful information.

Because participants were aware of word changes, there is a possibility that they might have adopted artificial strategies that differ from normal reading, and these strategies could have affected the results. We assume that any such strategies would have developed over the course of the experiment, which would result in an interaction between our experimental factor and the ordinal position of the trials in the experiment. We therefore conducted one extra analysis treating trial number as a predictor in the model of go-past times in the *sentence 2* region. Trial number was centered, and entered in interaction with condition, both as a fixed effect and in the random effect structure. The main effect of Trial was significant, with a negative coefficient, indicating that participants generally sped up in their reading during the course of the experiment (β = −0.007, *t* = −4.59). However, trial did not significantly interact either with the plausibility contrast (β = 0.002, *t* = 1.18) or with the change-facilitation contrast (β = 0.001, *t* = 0.49). Moreover, both the plausibility effect (β = −0.208, *t* = −6.92) and the change facilitation effect (β = −0.168, *t* = −7.09) remained significant with the addition of the Trial factor. Thus, although trial number predicted the baseline reading speed of participants, the size of both the plausibility effect and the change-facilitation effect remained roughly constant throughout the experiment. Thus, we could not find evidence that the eye-movement results were affected by the adoption of artificial strategies that developed through the course of the experiment.

## 3. Discussion

In the beginning of this paper, we posed several questions about regressions; what is the role of regressions, when do they take place, and how can this process be measured? Previous research indicated that regressions facilitate comprehension (Schotter et al., 2014), that information processed during regressions leads to memory update (Booth and Weger, 2013), and that readers fixate linguistically relevant information during regressions (Mitchell et al., 2008; von der Marlsburg and Vasishth, 2011, 2013). All of these previous observations suggest that regressions serve a greater purpose than the mere delay of subsequent input that would be predicted by the *Time Out Hypothesis* (Mitchell et al., 2008).

The study that we report here provides further evidence that information that is processed during regressive episodes is recruited in the language comprehension process. Specifically, we show that lexical-semantic information is actively processed during regressions and is used to recover from processing difficulty. We show that this information is used on-line, while the remaining stimulus is being read. Thus, our study goes further than that of Schotter et al. (2014) and Booth and Weger (2013), who use off-line post-trial comprehension questions as evidence for the effect of regressions on comprehension accuracy and memory. We note, however, that although we found evidence for facilitation in on-line measures, this effect was not immediate. The earliest possible point where we might have detected a change facilitation effect is in the go-past time measure on the critical word. However, go-past time showed no strong evidence for a facilitation effect at any position within the first sentence (although there was a numeric effect in the predicted direction on the final region of Sentence 1, which may indicate that more power is needed to detect this effect). Instead, there was strong evidence for facilitation in the second sentence, where the means for the change condition approached those of the plausible condition for both go-past time and proportions of regressions, while the implausible condition continued to show evidence of considerable processing difficulty. Thus, although lexical semantic information is indeed gathered during regressions, it may take some time for this information to be integrated in such a way that it can facilitate processing. If this is correct, it would be analogous to the situation with syntactic garden path sentences, where it has been shown that the initial commitment to the meaning of a locally ambiguous sentence can linger after this meaning becomes incompatible with the continuation of the sentence (Christianson et al., 2001; Sturt, 2007; Slattery et al., 2013). In both cases, this suggests that large changes to the meaning of a sentence can take time to be integrated into the developing interpretation.

Although our study was not designed to provide a detailed test of theories of the function of regressions, our results fit with the idea that one of the purposes of regressions is to check the previous context for potentially mis-perceived input given contextual misfit (Levy et al., 2009; Slattery, 2009; Bicknell and Levy, 2011). In particular, Bicknell and Levy (2011) (see also Bicknell and Levy, 2010) describe what they call the “confidence falling” account of regressions: “The model proposes that when a new word fits relatively poorly with what the reader believed the prior context to be, and relatively better with an alternative visually similar possibility, the reader's confidence in the identity of the prior context will be reduced. In this situation, it becomes useful to get more visual information about the prior context, and thus make a between-word regression” (Bicknell and Levy, 2011, p. 932). These predictions, thus, are compatible with high level of regressions in the current experiment where the critical word in the change and implausible conditions was not compatible with the previous context, and a visually similar alternative with a higher frequency to a pre-change context word improved the semantic fit. Under these conditions, the main purpose of the regressions would be to confirm whether mis-perception has taken place, by checking the previous context to verify whether the word is the initially encoded one, or a visually similar one. Our *change* condition simulates the situation where this check confirms the existence of the visually similar word, and processing then continues with greater confidence about the context. Further work should manipulate the visual similarity of words in the context, to investigate this idea further.

A second aspect of our results that deserves comment is the plausibility effect. We found inflated first fixation durations for the implausible condition relative to the plausible condition in the first fixation duration at the critical word. This represents the earliest point in the eye-movement record that the effect can be measured, confirming that the relevant information is recruited very rapidly in the incremental comprehension process. Our results are thus compatible with Warren and McConnell (2007), who found elevated first fixation durations on the target word for sentences that described physically impossible events (e.g., *The man used a photo to blackmail the thin spaghetti yesterday evening*). In our judgement, the sentences that we used in our own implausible condition also mostly describe physically impossible events, and thus confirm that such incongruities are registered immediately by the cognitive system. Indeed, Matsuki et al. (2011) have found first-fixation effects even for milder incongruities, in sentences that describe physically possible (though improbable) events (e.g., *Donna used the hose to wash her filthy hair after she came back from the beach*).

Before concluding this part of the discussion, it is worth noting that predictability is also a factor that can affect the eye-movement record, in early measures such as first fixation (see Rayner et al., 2011; Staub, 2011, inter alia). As pointed out by Matsuki et al. (2011), it is often difficult to separate the influence of predictability from that of plausibility, unless both variables are controlled or manipulated in the design. Predictability was not a focus of the current study, and as we did not manipulate or control this variable, we acknowledge that our plausibility effect may have included a predictability component.

Finally, a major goal of the current study was to investigate the feasibility of using the reverse boundary change paradigm for studying regressions, in the context of recovery from processing difficulty. We implemented a relatively simple design with a strong plausibility manipulation, in a situation where we believed that high levels of regressions would be observed in the relevant conditions. Within these conditions, we did observe high levels of regressions in the implausible and change conditions, and crucially, we confirmed that a robust change-facilitation effect could be measured. This provides initial evidence for the feasibility of the method, paving the way for further studies, where more nuanced theoretical questions might be studied.

To conclude, we have reported an eye-tracking study showing that lexical semantic information is processed in regressions, and is used in recovery from processing difficulty. At the same time, the study demonstrates the feasibility of using the reverse boundary change paradigm for future studies examining more nuanced theoretical questions.

## Ethics statement

This study was carried out in accordance with the recommendations of the British Psychology Society. The protocol was approved by the Psychology Research Ethics Committee at the University of Edinburgh. All subjects gave written informed consent in accordance with the Declaration of Helsinki.

## Author contributions

PS conceptualized the study, prepared the stimuli, supervised the running of the experiment, analyzed the results, and led the writing of the report. NK collaborated in conceptualizing the study, and in writing up the results.

### Conflict of interest statement

The authors declare that the research was conducted in the absence of any commercial or financial relationships that could be construed as a potential conflict of interest.

## Footnotes

^1^https://blogs.umass.edu/eyelab/software/

^2^This figure is based on 14 participants (due to a coding error, completion times for the display change were only recorded for the final 14 participants, although initiation times for the change were recorded for all participants).

^3^This could occur if the display change is triggered by a so-called “hooked” saccade, where the reader's gaze momentarily crosses the boundary during a saccade, but then moves a short distance to the left before landing on the word before the boundary.

^4^A subsidiary analysis was also conducted in which trials were deleted from the change condition when the display change was initiated following the onset of a fixation (there were 80 such trials). However, the results were almost identical in all relevant respects to those reported below, so we do not include that analysis here. Note that in our version of the boundary change paradigm, the display change involves a very minimal change of visual information in a word that is positioned at least two words to the left of the current gaze position. This is in contrast to the traditional boundary change paradigm, where display changes may occur on a word while it is currently being fixated (see Slattery et al., 2011, for a discussion of the consequences of display changes during a fixation under these conditions).

^5^Although we display means for four regions in Figure 2, we provide statistical analysis only for the context word. This is to reduce the number of statistical comparisons made.

^6^Many participants reported a range of proportions, in which case, we use the mid-point of the range as that participant's estimate.
