# Supplementary material for: Processing Information During Regressions: An Application of the Reverse Boundary-Change Paradigm
Source: Front Psychol. 2018 Sep 4;9:1630. doi: 10.3389/fpsyg.2018.01630 (PMC6132172; doi:10.3389/fpsyg.2018.01630)
Supplement: Supplementary file 1 [file Data_Sheet_1.pdf]

Appendix

The 48 experimental sentences are shown below. In each sentence, the critical word is marked with an asterisk, and the plausible and implausible variants of each item are separated with a slash, with the plausible variant appearing first. In the change condition, the implausible variant changed to the plausible variant at the point where the reader's gaze crossed an invisible boundary, which was located at the left edge of the space immediately preceding the critical word. In all cases, the first sentence was presented on one line, and the second sentence was presented on the next line.

1. The journalist knew that his ankle/angle would be very sore* after the strenuous gym class. It had happened a few times before.
2. Apparently, the cornea/corner had been injured* during the boxing match that evening. The medical team had been very professional.
3. She went to the shop to buy a new cloak/clock that she would wear* at the neighbour's Halloween party. It looked very impressive indeed.
4. Apparently, the new decree/degree was used to prevent* protest marches against the government. These had been causing some concern.
5. The brand new choir/chair had a better sound* after the change of conductor. Apparently he was very enthusiastic.
6. She said that her favourite robe/role had some stains* on it from the red wine. This was really quite annoying.
7. I heard that the fiend/field had a new plan* to perform some wicked deeds. Things could get very evil indeed.
8. The teacher explained that the squire/square of the knight* was quite powerful in his district. It was a mainly agricultural community.
9. The young athlete's skull/skill had been fractured* in the accident at the gym. One has to be very careful.
10. Unfortunately, the shack/shock that they had built* had no legal planning permission. It was fairly typical in this district.
11. Because of the heavy snowfall, the brunch/branch had to be postponed* for two or three weeks. Nobody was particularly surprised.
12. Because the plank/plant was nearly broken*, the carpenter came to replace it. It was a fairly straightforward job.
13. I could see that the scalp/scale had lots of dandruff* that needed some serious attention. Perhaps the doctor should be informed.
14. The police knew that the dagger/danger had been purchased* by their prime suspect last month. A receipt had been found in the car.
15. Yesterday, the novice/notice surprised everyone by winning* first prize in the race. This certainly hadn't been predicted by anyone.
16. According to the teacher, the clash/class took place between enemies* who really hated each other. This kind of thing happened occasionally.
17. She heard the stork/story that was flapping* its wings noisily outside the window. The whole situation was bizarre.

18. John was glad about all the spice/space that could be sprinkled* into the casserole for dinner. He enjoyed this style of cooking.
19. John described an interesting plate/place that had shattered* into tiny pieces on the floor. Apparently he told lots of people about it.
20. Kevin realized that the new stove/store was already broken* and he demanded a full refund. This was the least that could be expected.
21. He swept the flour/floor that had been spilled* in the kitchen before putting the bread in the oven. Mondays were often a bit hectic.
22. The weather forecaster said that the frost/front would probably melt* by the middle of the afternoon. The forecast was based on a new computational model.
23. The scientist pointed out that the birch/birth had actually been planted* in the forest years ago. However, not many people were interested.
24. The troops were slowed down by the marsh/march that was blocking* their path to the final destination. Geographical information is important in military campaigns.
25. The soldiers were discussing the manor/major that had been built* near their barracks outside town. They were just passing the time of day.
26. The settlers came across a rider/river that had dismounted* his horse and was approaching them. It wasn't always possible to trust strangers.
27. The doctor thought that the bread/break had been baked* with too much sugar and salt. Health is always an important consideration.
28. The crowd was shocked when the noose/noise had become untied* and the condemned man quickly escaped. It took about four days to find him.
29. In many people's opinion, no truce/trace would ever be negotiated* until the diplomats had met. The negotiations would be tough.
30. Apparently, the stroke/strike was caused by diabetes* and several other health issues. Always follow your doctor's advice.
31. According to my friend, the resort/report had been built* in the 1930s during the depression. It was beginning to show its age.
32. They thought that the snort/sport that had startled* them came from behind a large tree. The forest is quite scary in the evening.
33. The teacher said that the worm/word was used as bait* for fishing in the sea. The boat would be ready the next week.
34. I was told that the valve/value was very rusty* because of the humid sea air. Many people claimed the same thing.
35. It turned out that the pair/pain had been breeding* in their native habitat for years. Apparently they were an endagered species.
36. We heard that the foam/form was used for protecting* the valuable merchandise during shipping. The last thing you want is a customer complaint.
37. He told them that the storm/story could easily reach hurricane* force and cause structural damage. However, the forecast was based on faulty information.

38. Laura thought that the shoe/show was slightly too tight* to be comfortable to wear. There would be quite a lot of walking involved.

39. The salesman knew that the prize/price could easily be won* by somebody with business flair. Everybody was working so hard.

40. We all agreed that that the ascent/accent was really quite exhausting* due to lack of oxygen. However, the view from the top was great.

41. It was quite clear that the beard/board really needed shaving* to make it suit his face. Otherwise, he would just look untidy.

42. We all noticed that the coil/coal had an interesting spiral* shape like a snail shell. Nature can be very beautiful sometimes.

43. Nobody guessed it but the cork/cook was in the bottle* all along and nobody knew. Everybody was a bit drunk.

44. There was an old horse/house that John had ridden* when he was a boy. It couldn't run fast any more.

45. Jane thought that the pork/park had clearly been cooked* with a lot of spices. She wanted to know the recipe.

46. Towards evening, we spotted a moose/mouse that had brown antlers* and it was running around. This is quite an interesting animal.

47. John was disappointed by the decay/delay that affected his tooth* and had been found by the dentist. He had regularly brushed his teeth.

48. After a while, we found that the alloy/alley was very slightly magnetic* and also was extremely heavy. We did some more tests the next day.
